# steve bAccumulation of nerve growth factor and its receptors in the uterus and dorsal root ganglia in a mouse model of adenomyosis

**DOI:** 10.1186/1477-7827-9-30

**Published:** 2011-03-08

**Authors:** Yan Li, Shao-fen Zhang, Shi-en Zou, Xian Xia, Lei Bao

**Affiliations:** 1 Department of Gynaecology, Obstetrics and Gynaecology Hospital, Fudan University, Shanghai, 200011, PR China; 2 Reproductive medicine center, Ruijin Hospital, Shanghai Jiaotong University School of Medicine, Shanghai, 200025, PR China

## Abstract

**Background:**

Adenomyosis is a common gynecological disease, which is accompanied by a series of immunological and neuroendocrinological changes. Nerve growth factor (NGF) plays a critical role in producing pain, neural plasticity, immunocyte aggregation and release of inflammatory factors. This study aimed to investigate the expression of NGF and its two receptors in uteri and dorsal root ganglia (DRG) in an adenomyosis mouse model, as well as their relationship with the severity of adenomyosis.

**Methods:**

Forty newborn ICR mice were randomly divided into the adenomyosis model group and control group (n = 20 in each group). Mice in the adenomyosis model group were orally dosed with 2.7 μmol/kg tamoxifen on days 2-5 after birth. Experiments were conducted to identify the expression of NGF- beta and its receptors, tyrosine kinase receptor (trkA) and p75 neurotrophin receptor (p75NTR), in the uterus and DRG in four age groups (90+/-5 d, 140+/-5 d, 190+/-5 d and 240+/-5 d; n = 5 mice in each group) by western bolt, immunochemistry and real time reverse transcription-polymerase chain reaction.

**Results:**

Adenomyosis, which became more serious as age increased, was successfully induced in dosed ICR mice. NGF-beta, trkA and p75NTR protein levels in the uterus and trkA mRNA levels in DRG were higher in the older aged adenomyosis model group than those in controls (190+/-5 d and 240+/-5 d groups, P < 0.05). The expression of NGF-beta and its receptors in the uterus increased gradually as age increased for adenomyosis mice (190+/-5 d and 240+/-5 d, P < 0.05, compared with 90+/-5 d) but it showed little change in control mice. The mRNA level of trkA in DRG also increased as age increased in the adenomyosis model group (190+/-5 d and 240+/-5 d, P < 0.05, compared with 90+/-5 d) but was unchanged in controls. The mRNA level of p75NTR in DRG was not different between the adenomyosis and control groups and was stable from young to old mice.

**Conclusions:**

NGF- beta can be used as an indicator for the severity of adenomyosis. The gradually increasing level of NGF- beta and its receptors while the disease becomes more severe suggests an effect of NGF- beta on pathogenic mechanisms of adenomyosis.

## Background

Adenomyosis is defined as the presence of ectopic endometrial glands and stroma within the myometrium. It is a relatively frequent endomyometrial pathology that is found in multiparous women between 40 and 50 years of age [1]. Approximately 2/3 of women with adenomyosis are symptomatic with menorrhagia and dysmenorrhoea [2]. However, the etiology and pathogenic mechanisms responsible for adenomyosis are poorly understood.

An impaired neuroimmune status may be necessary for the maintenance of adenomyosis, and it could play an important part in the mechanism by which the symptoms of adenomyosis are generated [3-5]. NGF was the first neurotrophic factor to be discovered and it plays a critical role in producing pain, neural plasticity, immunocyte aggregation, and release of inflammatory factors [6,7]. Several studies have suggested that abnormal expression of NGF in adenomyosis and endometriosis might be involved in the pathomechanism of these diseases [8-10]. Studies in humans and rat have shown that there is an intense NGF-β immunoreactivity near ectopic endometriotic glands [10-12]. The receptors of NGF are high-affinity tyrosine kinase receptor (trkA) and low-affinity p75 neurotrophin receptor (p75NTR), and they are immunoreactive in nerve fibers that are close to endometriotic glands and blood vessels [13-15]. A potential involvement of NGF-β in innervation and pain occurring in endometriosis has been suggested [9]. In our previous studies, we found a higher NGF-β expression in adenomyosis mice, which might explain the augmented innervation and inflammation status in the uterus [8]. Abnormal expression of the nonfunctional subunit NGF-α (not the functional subunit NGF-β) has also been detected in uteri of newborn mice, which acquire adenomyosis in adulthood [16,17].

Discovered over 50 years ago, NGF has been extensively studied in various biological systems in an attempt to understand its role in adenomyosis. It has been shown to play significant roles in the transmission of physiological and pathological pain [6,7]. Transgenic animals lacking the gene for NGF and trkA are hypoalgesic and are born without small diameter nociceptive sensory neurons [18,19]. In contrast, animals that over express NGF, or humans injected with NGF in cutaneous targets, have behavioral or sensorius hyperalgesia [20,21]. The mechanism of how NGF is involved in pain is manifold and complicated. NGF can promote the proliferation of nerve fibers and improve their sensitivity. NGF is crucial for the development of sympathetic and small fiber sensory neurons that serve as nociceptors [6]. It is also involved in sensitizing or exciting the terminals of sensory nerve fibers by stimulating the expression and release of neuropeptides [22]. NGF plays an important role in mediating and/or regulating the immune response. It has been proposed that NGF is involved in the recruitment, survival, and activation of mast cells, T cells of the Th2 phenotype, and eosinophil infiltration [23]. By stimulating the degranulation of mast cells, NGF can promote the release of inflammatory mediators such as serotonin [24]. In addition, NGF affects non-neurocyte proliferation, such as in myogenous cells [25], cystic smooth muscle cells [26] and corneal epithelial cells [27]. NGF might also participate in stress-triggered and substance P-mediated abortion [28].

Adenomyosis is associated with recurrent and aggressive pain syndrome, impaired immune status, and also characterized by intensified weighted uterus and a high rate of spontaneous abortion [2-4,29]. Since NGF has abnormal expression in adenomyosis and has multiple effects on innervation, immunoregulation, cell proliferation and production of pain, it might be involved in the pathomechanism of adenomyosis. If NGF plays a role in adenomyosis, NGF levels and/or its receptors might change with the progress of this disease. To determine this possibility, we administered tamoxifen in an adenomyosis mouse model. This model has an aggravated pathogenetic condition of adenomyosis as age increases. In addition, it is not associated with trauma and has relatively normal sex hormone levels, which are important for considerations because trauma and sex hormone affect the expression of NGF [6,7,30].

In this study, we examined the relationship between NGF-β and the severity of adenomyosis. By examining the protein and mRNA levels of NGF and its receptors in the uterus and dorsal root ganglia (DRG) at different ages in adenomyosis mice, we aimed to evaluate any changes with the severity of adenomyosis in peripheral organs and the central nerve system.

## Methods

### Animals and treatments

Neonatal healthy female ICR mice (SCXK 2007-0005, SLAC laboratory animal CO. LTD, Shanghai, China) were housed in negative pressure isolators with a controlled temperature (23-25°C) and 12 h alternating light (8 a.m to 8 p.m) and dark cycle. To induce the adenomyosis mouse model, neonatal mice were orally dosed daily on days 2 to 5 after birth (day of birth was day 1) with 2.7 μmol/kg tamoxifen (Fudan Forward Co., Shanghai, China) suspended in a peanut oil/lecithin/condensed milk mixture (2:0.2:3, by volume) at a dose volume of 5 μl/g body weight. Controls received no treatment. The 4 day estrous cycle was determined by the presence of a characteristic vaginal discharge in the morning. ICR Mice with at least 1 consecutive 4-day estrous cycle were used in the present study. All experimental procedures were approved by the Ethics Committee of Obstetrics and Gynecology Hospital, Fudan University.

### Euthanasia and processing of tissue

All mice were sacrificed at 5 p.m. by injecting propofol (10 mg/kg injection, i.v.) on proestrus at age 90 ± 5, 140 ± 5, 190 ± 5 and 240 ± 5 d (n = 5 in each group). T13 to L2 DRG were harvested and stored in liquid nitrogen. Bilateral uteri were removed. After weighing, the uteri were either frozen in liquid nitrogen or fixed in 4% neutral paraformaldehyde at 4°C.

### Immunohistochemistry and immunofluorescence

Formalin-fixed, 5 μm paraffin sections of mouse uterus were dewaxed and taken to water by immersing in xylene and gradient ethanol. Hematoxylin and eosin (HE) staining was performed first to determine whether there were adenomyosis nodes. Sections were then immersed in boiling citrate buffer (pH 6.0) for 20 min. Endogenous peroxidase activity was blocked with 3% H2O2 for 30 min and then goat serum for 15 min. Sections were then incubated with specific polyclonal rabbit antibodies for NGF (Santa Cruz Biotechnology, Santa Cruz, CA, USA) at a dilution of 1:500, p75NTR (Epitomics, Burlingame, CA, USA) at a dilution of 1:100, or trkA (Epitomics, Burlingame, California, USA) at a dilution of 1:100, at 4°C overnight. The next day, sections were incubated with biotinylated goat anti-rabbit IgG (Zhongshan Golden Bridge Biotechnology Co., Ltd, Beijing, China) for 15 min and then streptavidin-peroxidase complex (Zhongshan Golden Bridge Biotechnology Co., Ltd) for another 15 min at room temperature. Staining was visualized with 3,3'-diaminobenzidine for 3 min at room temperature (Zhongshan Golden Bridge Biotechnology Co., Ltd) and hematoxylin counter stain. After incubated with trkA (1:100) at 4°C overnight, some sections were incubated with biotinylated goat anti-rabbit IgG (Boster, Wuhan, Hubei, China) for 30 min at 37°C and then streptavidin- fluorescein isothiocyanate complex (Boster, Wuhan, Hubei, China) for another 30 min at 37°C. Negative controls included omission of the primary or secondary antibody. There was no labeling in different groups and any of the control sections. Paraffin sections of mouse brain were used for positive control following the instruction for the antibody of NGF, p75NTR and trkA. Slides were examined using a radiophoto microscope (BX51, Olympus, JP) equipped with a digital camera (DP71, Olympus, JP).

### Western blot analysis

The uteri of adenomyosis and control mice were homogenized and lysed in ice cold RIPA lysis buffer (consisting of 50 mM Tris [pH 7.4], 150 mM NaCl, 1%NP- 40, 0.5% sodium deoxycholate, 0.1% SDS and protease inhibitors; Beyotime, Haimen, Jiangsu, China) with 1 mM phenylmethylsulfonyl fluoride. Each sample containing 40 μg protein was separated by 12% SDS-PAGE. Protein bands of samples were then electrically transferred on PVDF membranes (Millipore, Billerica, MA, USA) at 300 mA for 2 h. After blocking with 10% defatted milk, membranes were incubated with rabbit multiclonal anti-mouse NGF (Santa Cruz) at a dilution of 1:500, monoclonal anti-mouse trkA (Epitomics) at a dilution of 1:1000, monoclonal anti-mouse p75NTR (Epitomics) at a dilution of 1:5000, or monoclonal anti-mouse beta-actin (Sigma, St. Louis, MI, USA) at a 1:1000 dilution at 4°C overnight. Membranes were then incubated with peroxidase-labeled secondary antibody (Jackson, West Grove, PA, USA) at a dilution of 1:5000 for 1 h. The chemiluminescent signal was developed using BeyoECL Plus (Beyotime, Haimen, Jiangsu, China) for 2 min and quantitated over a 5-10 min exposure using a Chemiluminescent and Fluorescent Imaging System (Fluochem FC2, Alpha Innotech, San Leandro, CA, USA).

### Real time reverse transcription polymerase chain reaction (real time RT-PCR)

Total RNA from DRG (T13 to L2 together) was isolated using TRIzol reagent (Invitrogen, Carlsbad, CA, USA). Complementary DNA was synthesized from 1 μg RNA using superscript II reverse transcriptase and oligo (dT)12-18 primers (Fermentas, Glen Burnie, MD, USA). Real-time PCR reactions were performed using a Bio-Rad IQ5 machine (Bio-Rad, Hercules, CA, USA) and SYBR Premix Ex TaqTM II (Takara, Dalian, Liaoning, China) according to the manufacturer's instructions. Each 20 μl reaction buffer contained 10 μl of 2×SYBR Premix Ex TaqTM II, 0.8 μl of primers at 10 μM, 2 μl of diluted cDNA template, and 6.4 μl of ddH2O. The PCR conditions were 95°C for 5 sec and 60°C for 30 sec, for 40 cycles. The primers for *Actb *(gene for beta-actin) were: forward, 5'-CCT CTA TGC CAA CAC AGT GC-3' and reverse, 5'-GTA CTC CTG CTT GCT GAT CC-3'; *Ntrk1 *(gene for trkA): forward, 5'-ATA TCT AGC CAG CCT GCA CTT TGT-3' and reverse, 5'-TGC TCA TGC CAA AGT CTC CA-3'; and *Ngfr *(gene for p75NTR): forward, 5'-TAT AGA CTC CTT TAC CCA CG-3' and reverse, 5'-AAT GTC AGC TCT CTG GAT G-3'. The relative mRNA levels of *Ntrk1 and Ngfr *were calculated and normalized against the level of *Actb*.

### Statistics

All data were analyzed by SAS 6.12 (SAS institute Inc, Cary, NC, USA) and expressed as mean ± SD. The results of wesrern blot and gene expression were subjected to one-way ANOVA. The results of uterine and body weight were subjected to t-tests to compare the mean value. A value of P < 0.05 was considered to be statistically significant.

## Results

### Adenomyosis model of ICR mice

**Figure 1:**
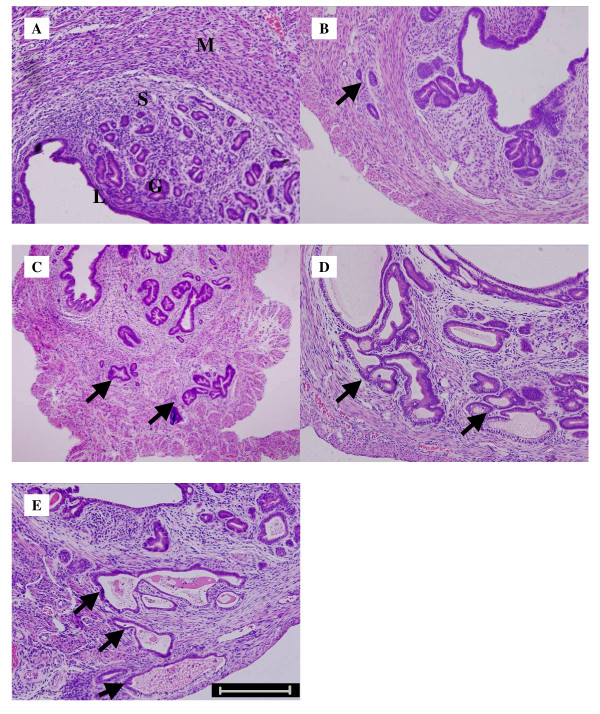
**HE staining of paraffin-embedded sections**. A: Uterus of 140 ± 5 d ICR mice as control. B:Uterus of 90 ± 5 d ICR mice with biopsy confirmed adenomyosis; C: Uterus of 140 ± 5 d ICR mice with biopsy confirmed adenomyosis; D: Uterus of 190 ± 5 d ICR mice with biopsy confirmed adenomyosis; E: Uterus of 240 ± 5 d ICR mice with biopsy confirmed adenomyosis; (L, luminal epithelium; G, glandular epithelium; S, stromal cell; M, myometrium cell; arrows, ectopic endometrium; Bar = 50μm).

All mice dosed with tamoxifen developed adenomyosis in this study. None of the controls had adenomyosis nodes in the uteri (Figure 1A). The depth and area of ectopic endometrium were increased as age increased for tamoxifen dosed mice (Figure 1B-E). Nodules of endometrial glands and stroma were present deep within the myometrium, sometimes extending to, but not penetrating, the serosa. Unilateral uterine weight and body weight for both dosing mice and controls is shown in Table 1. For tamoxifen dosed mice, at first, their uteri were lighter (90 ± 5 d, P < 0.05) and then they became heavier (190 ± 5 d, 240 ± 5 d; P < 0.05) than those in controls.

**Table 1:** The unilateral uterine weight and body weigh of adnomyosis and control ICR mice

Group	Age (d)	Uterine weight (mg)	Body weight (g)
Control	90 ± 5	227.6 ± 23.5	43.80 ± 2.35
	140 ± 5	227.8 ± 17.2	43.32 ± 2.62
	190 ± 5	215 ± 9.76	45.98 ± 2.92
	240 ± 5	223.8 ± 24.2	44.17 ± 3.12
Adenomyosis	90 ± 5	174.8 ± 14.8*	42.78 ± 2.49
	140 ± 5	206.0 ± 21.0	47.84 ± 3.21
	190 ± 5	243.3 ± 21.6*	49.68 ± 3.50
	240 ± 5	243.0 ± 27.0*	45.84 ± 5.11

MEAN ± SD; n = 5; *P < 0.05 compared with the control group

### Immunochemical localization of NGF-β, trkA and p75NTR in the uterus

**Figure 2:**
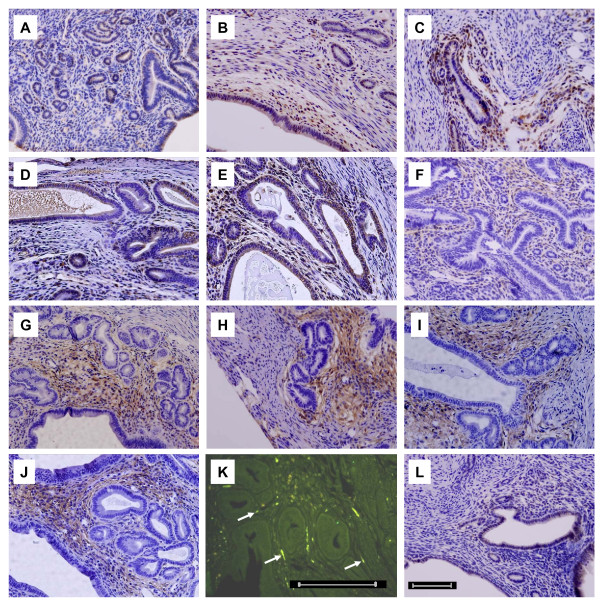
**Immunostaining of NGF-β, p75NTR and trkA in uteri of ICR mice with adenomyosis or not**. A: Uterus of 140 ± 5 d control ICR mice stained for NGF-β. B-E: Immnostaining of NGF-β in uterus of 90 ± 5 d, 140 ± 5 d, 190 ± 5 d and 240 ± 5 d ICR mice with adenomyosis, respectively. F: Uterus of 140 ± 5 d control ICR mice stained for p75NTR. G-J: Immnostaining of p75NTR in uterus of 90 ± 5 d, 140 ± 5 d, 190 ± 5 d and 240 ± 5 d ICR mice with adenomyosis, respectively. K-L: trkA immnoreactivity was detected in nerve fibers (K; arrows, nerve fibers), luminal and glandular epithelial cells of endometrium (L) in uterus of adenomyosis ICR mice aged 140 ± 5 d. (Bar = 100 μm).

We performed immunohistochemical and immunofluorescence analysis to analyze the temporal and spatial changes in the expression of NGF-β, trkA and p75NTR in the uterus. In the uteri of both adenomyosis and control mice, NGF-β immunoreactivity was predominantly observed in the luminal epithelial cells, glandular cells, and stromal cells of the endometrium. NGF-β immunohistochemical positive signals were scarce in myometrial cells (Figure 2A-E). Immunoreactivity of p75NTR was detected mainly in endometrial stromal cells (Figure 2F-J). Immunoreactivity of trkA was detected in nerve fibers distributed in the uterus (Figure 2K). Additionally, trkA was also weakly expressed in luminal epithelial cells and glandular cells in the endometrium (Figure 2L). The ectopic endometrium of adenomyosis mice had a similar immunolocation as eutopic endometrium.

### Western blot analysis of NGF-β, proNGF and its receptors in the uterus

**Figure 3:**
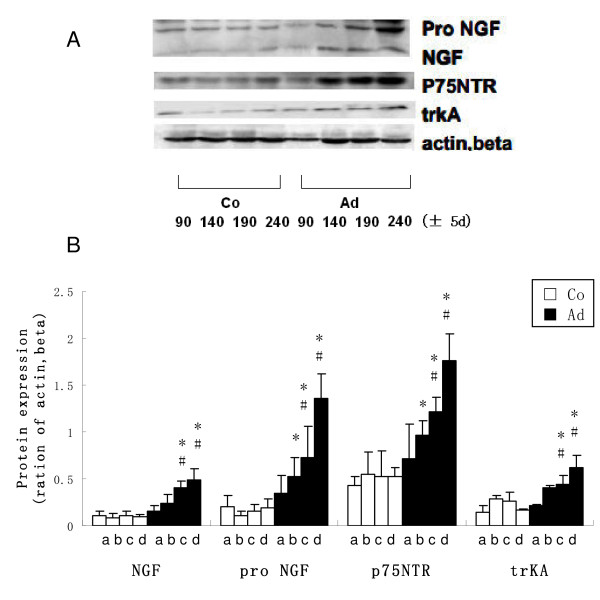
**Western-blot analysis of NGF-β, pro NGF, p75NTR and trkA in adenomyosis (Ad) and control (Co) ICR mice uteri**. A: Representative Western-blot analysis of NGF-β, pro NGF, p75NTR and trkA in adenmoysis and control mice in different age groups. B: Densitometirc analysis of NGF-β, pro NGF, p75NTR and trkA in adenomyosis and control mice in different age groups expressed as a percentage after actin, beta normalization (n = 5; a = 90 ± 5 d, b = 150 ± 5 d, c = 190 ± 5 d, d = 240 ± 5d; *P < 0.05, compared with the control mice with the same ages; #P < 0.05, compared with the 90 ± 5 d mice with the same treatment).

The protein levels of NGF (13 kDa) and its precursors (proNGF, 27 kDa), p75NTR (75 kDa) and trkA (145 kDa), in the uteri of adenomyosis and control mice were detected by western bolts (Figure 3). NGF, pro NGF, p75NTR and trkA levels increased in the uteri of adenomyosis mice as age increased (190 ± 5 d and 240 ± 5 d, P < 0.05, compared with 90 ± 5 d) and remained unchanged in controls. The expressions of NGF, its precursors and receptors of adenomyosis mice were higher than those in controls in the 190 ± 5 and 240 ± 5 d age groups (P < 0.05).

### Gene expression of trkA and p75NTR in the DRG

**Figure 4:**
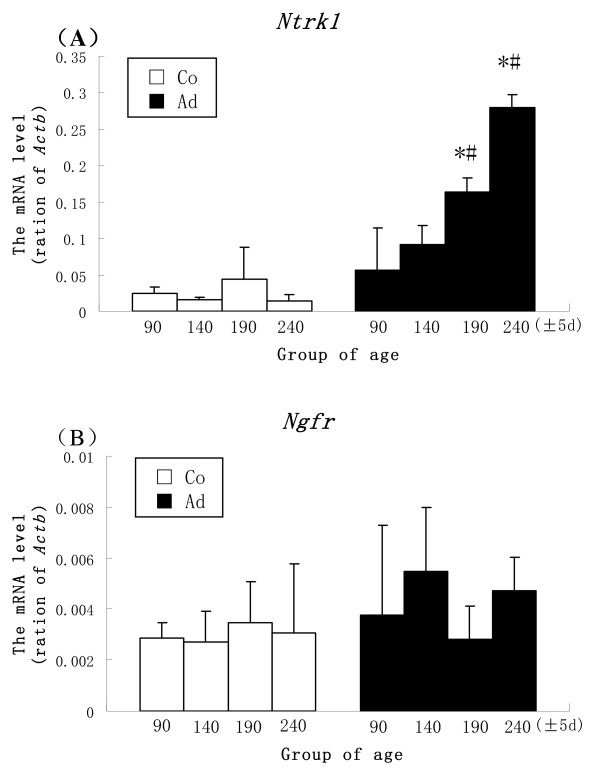
**mRNA expression of *Ntrk1 *and *Ngfr *in DRGs of adenomyosis (Ad) and control (Co) mice**. A: the mRNA level of *Ntrk1 *in DRGs of adenomyosis and control mice for different age groups. B: the *Ngfr *level in DRGs of adenomyosis and control mice for different age groups. (n = 4; *P < 0.05, compared with the control mice with the same ages; #P < 0.05, compared with the 90 ± 5 d mice with the same treatment).

The mRNA expressions of *Ntrk1 *(gene of trkA) and *Ngfr *(gene of p75NTR) in DRG of adenomyosis and control ICR mice in the different age groups were examined by real time RT-PCR. *Ntrk1 *levels were significantly increased in adenomyosis mice compared with those in controls (190 ± 5 d and 240 ± 5 d, P < 0.05). *Ntrk1 *levels increased as age increased in adenomyosis mice (190 ± 5 d and 240 ± 5 d, P < 0.05, compared with 90 ± 5 d). The mRNA levels of *Ngfr *were not different between adenomyosis and control mice and remained stable as age increased (Figure 4).

## Discussion

In this study, we investigated the expression of NGF and its receptors (p75NTR and trkA) in the uterus and DRG of mice with adenomyosis. A mouse model with progressively worsening adenomyosis was generated to study the relationship between the expression of NGF and the severity of adenomyosis. We showed that NGF-β, p75NTR and trkA protein levels in uteri and trkA mRNA levels in DRG were higher than those in controls, while they gradually increased as adenomyosis worsened.

We used tamoxifen administration to create the adenomyosis mouse model. The precise mechanism for this mouse model has not been fully clarified. It is proposed that paracrine signaling prevents differentiation of uterine myocytes in the mesenchyme, and over a period of time this may permit downgrowth of the endometrium into the myometrium [16]. In previous studies, the area and depth of the ectopic endometrium in uteri of an adenomyosis mouse model constantly increased as age increased [17,31]. In the present study, we showed a progressively infiltrated endometrium (Figure 1) and increased uterine weight (Table 1) in adenomyosis mice. In mice administered with tamoxifen, initially their uteri were lighter, which might have been the result of depauperate mesenchyme [17] and then they became heavier, which might have been because of ectopic endometrium proliferation [2], compared with controls. We chose four progressive age groups to represent four stages of adenomyosis.

In the present study, immunoreactivity of NGF-β was predominantly detected in the luminal and glandular epithelial cells, and stromal cells of the endometrium. We observed p75NTR mainly in stromal cells of the endometrium and trkA in nerve fibers as well as in luminal and glandular epithelial cells. The immunolocation of NGF-β, p75NTR and trkA in the ectopic endometrium was similar to eutopic endometrium in adenomyosis mice. Our finding of NGF's location is consistent with previous studies [32]. However, beside nerve fibers [12,15], we also found p75NTR and trkA immunostaining in endometrial cells. The expression of NGF and its receptors in functional cells of the uterus suggests a local effect, except for the well known effect in the nervous system.

We examined the abnormal increase of NGF and its receptors in uteri of adenomyosis mice by western blot. NGF-β, p75NTR and trkA protein levels in uteri gradually increased while age increased in adenomyosis mice, but they remained unchanged in control mice. In adenomyosis mice, as age increases, the disease progresses [17,31]. Therefore, NGF can be used as an indicator for the severity of adenomyosis. As a multi-functional cytokine and growth factor, over expression of NGF might exert an effect in multiple different ways. NGF is crucial for the development of sympathetic and small fiber sensory neurons that serve as nociceptors [6,7]. For adenomyosis, which also has an increased density of nerve fibers in the uterus of symptomatic patients [33], NGF might have the contribution to this abnormal innervation. NGF plays a crucial role in the generation of pain and hyperalgesia in several acute and chronic pain states [34,35]. NGF increases the excitability of primary afferents by altering ion channels or neurotransmitter production [36-38]. As a chemoattractant for granulocytes and mast cells, NGF strengthens immunoreactions and stimulates the degranulation of mast cells, which helps the release of pain-producing substances [39]. Therefore we speculate that this ever-increasing NGF effect in severe adenomyosis might contribute to the aggressive pain accompanied by this disease. NGF also has a relationship with uterine weight and participates in stress-triggered and substance P-mediated abortion [28,30]. These findings might be important for studies on adenomyosis-related hysterauxesis and spontaneous abortion.

The antibody we used for NGF-β (13 kD) can bind to proNGF (27 kD). As a precursor, proNGF reflected the reserve of mature NGF-β [40]. Pro-NGF can bind p75NTR with a high affinity and activate trkA, too [40,41]. In peripheral inflammation, pro-NGF is up-regulated and it plays a crucial role in inflammatory hypersensitivity [40]. Our findings regarding the increase of proNGF suggest that there is an abundant NGF-β store and that is has a biological effect on hypersensitivity in inflammation.

We observed that p75NTR was strongly expressed in endometrial stromal cells and weakly expressed in DRG, while trkA was strongly expressed in uterine nerve fibers and DRG but it was poorly expressed in luminal and glandular epithelial cells. These findings suggest that the stronger expression of p75NTR in the endometrium enables NGF to have a local effect on stromal cells of the uterus, while trkA might enable NGF to affect the peripheral and central nervous system. NGF could be taken up by nerve terminals and transferred to the central nervous system to exert its effect. We detected mRNA levels of NGF receptors but not NGF itself in DRG, which is not unexpected because NGF is not synthesized in this tissue [6].

The reason for the increase in NGF and its receptors in adenomyosis mice is unclear. The level of NGF-β and its receptors was mostly normal in tamoxifen administered mice at the ages of 90 ± 5 d and 140 ± 5 d for which ectopic endometrium was observed in the myometrium. This suggests that the abnormal expression of NGF-β is only an association but not the cause of adenomyosis. Estrogen promotes the expression of NGF in endometrium [42]. Adenomyosis has been shown to be an estrogen-dependent disease that has abnormal local estrogen production in the uterus [43,44]. Therefore, abnormal local estrogen levels might be responsible for the increase in NGF. It has also been reported that NGF is associated with a variety of autoimmune and inflammatory diseases (e.g. allergic diseases and asthma [45,46], and intestinal mucosa inflammation [47]) in which elevated NGF levels correlate well with the severity of the diseases. It is well known that adenomyosis is accompanied by various autoimmune phenomena in humans. For example, patients with adenomyosis have an increased number of macrophages, gamma delta T cells in ectopic and eutopic endometrium, and elevated preinflammatory factors and cytokines (e.g. TNF-α, IL-10, and IL-18) in the uterus and peritoneal fluid [29,48,49]. The augmentation of NGF might be the result of inflammatory status and then mediate and/or regulate the immune response. Therefore, we speculate that with the infiltration and growth of ectopic endometrium, uterine inflammation and local estrogen synthesis are increased, which could promote NGF synthesis. NGF might then participate in neural plasticity, local inflammation and production of pain. Therefore, it is possible that NGF might be one of the important factors that participate in the pathological mechanism of adenomyosis. In addition, it is worth mentioning that western blot and RT-PCR were performed from the whole uterus in this study. However, endometrial glandular, stromal, and myometrial components may differ in the expression of NGF and its receptors as the data of immunohistochemistry showed. So the increase of endometrial glandular and stromal in adenomyosis might affect the expression of NGF and receptors, too.

In the present study, we evaluated the expression of NGF, p75NTR and trkA in uteri and DRG of adenomyosis mice. The protein levels of NGF and its receptors in uteri and trkA mRNA levels in DRG were higher in adenomyosis mice compared with those in controls, and these levels increased with the severity of disease. Recently, a novel class of pain drugs that are based on antagonism of NGF have been investigated with a promising outcome [50]. Our results also suggest the possibility of anti-NGF therapy for curing adenomyosis pain; however, further study is required to determine this issue.

## Competing interests

The authors declare that they have no competing interests.

## Authors' contributions

YL has participated in study design, execution, analysis, manuscript drafting. SFZ has participated in study design, manuscript drafting and critical discussion. SEZ has participated in execution, analysis and critical discussion, XX has participated in execution and critical discussion, LB has participated in execution and analysis. All authors read and approved the final manuscript.
